# A Microscale Model for Combined CO_2_ Diffusion and Photosynthesis in Leaves

**DOI:** 10.1371/journal.pone.0048376

**Published:** 2012-11-07

**Authors:** Quang Tri Ho, Pieter Verboven, Xinyou Yin, Paul C. Struik, Bart M. Nicolaï

**Affiliations:** 1 Flanders Center of Postharvest Technology/BIOSYST-MeBioS, Katholieke Universiteit Leuven, Leuven, Belgium; 2 Centre for Crop Systems Analysis, Wageningen University, Wageningen, The Netherlands; Colorado State University, United States of America

## Abstract

Transport of CO_2_ in leaves was investigated by combining a 2-D, microscale CO_2_ transport model with photosynthesis kinetics in wheat (*Triticum aestivum* L.) leaves. The biophysical microscale model for gas exchange featured an accurate geometric representation of the actual 2-D leaf tissue microstructure and accounted for diffusive mass exchange of CO_2._ The resulting gas transport equations were coupled to the biochemical Farquhar-von Caemmerer-Berry model for photosynthesis. The combined model was evaluated using gas exchange and chlorophyll fluorescence measurements on wheat leaves. In general a good agreement between model predictions and measurements was obtained, but a discrepancy was observed for the mesophyll conductance at high CO_2_ levels and low irradiance levels. This may indicate that some physiological processes related to photosynthesis are not incorporated in the model. The model provided detailed insight into the mechanisms of gas exchange and the effects of changes in ambient CO_2_ concentration or photon flux density on stomatal and mesophyll conductance. It represents an important step forward to study CO_2_ diffusion coupled to photosynthesis at the leaf tissue level, taking into account the leaf's actual microstructure.

## Introduction

Photosynthesis is amongst the most important metabolic processes in plants. During photosynthesis, CO_2_ diffuses from the atmosphere into the leaf and finally to the site of carboxylation in the chloroplast stroma [1]. There is increasing evidence that diffusive resistances in the leaf are a limiting factor for photosynthesis [2], [3].

Fick's first law of diffusion has been used to describe the net CO_2_ flux from the external environment through the intercellular space towards the cells [4], [5]. It postulates that gas moves from places of high concentration to places of low concentration with a rate proportional to the gradient in concentration. The stomatal conductance (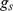) determines the gas exchange from the phyllosphere into the intercellular air space. The stomatal conductance for CO_2_ has been estimated based on the water vapour release from the leaf given the fact that water and CO_2_ share the same gaseous diffusion pathway [6], [7]. The mesophyll conductance (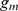) is defined as the conductance for the transfer of CO_2_ from the intercellular air space (*C*_i_) to the site of carboxylation in the mesophyll cells (*C*_c_). Both 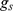 and 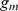 are apparent parameters rather than physical constants as they implicitly incorporate microstructural and biochemical features of the tissue, cells and organelles that are involved in the gas transport mechanism.

Several methods have been developed to estimate 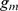. The most common method is to use a combination of gas exchange and chlorophyll fluorescence measurements [8], [9], [10], [11], [12]. It has been shown that 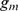 is sufficiently small to significantly decrease 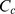, relative to 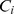, thereby limiting photosynthesis [1], [10], [13], [14], [15], [16], [17]. Many physiological and leaf microstructural features have been found to correlate with 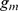, including photosynthetic potential [13], [17], [18], stomatal conductance [13], and mesophyll surface area exposed to intercellular air spaces [18]. Tholen and Zhu [3] showed that the resistances of the cell wall and chloroplast envelope were the most important cellular limitations to photosynthesis. Further, in early reports (e.g., [13]) 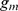 was considered constant for a given leaf at a given temperature. Recent evidence, however, suggests that 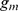 is variable [19], and a response of 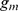 to CO_2_ and irradiance has indeed been found, resembling the response of 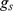 to CO_2_ and irradiance [1], [17]. The kinetics of change of 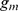 in response to CO_2_ have been demonstrated by observing the rate of change of 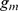 for different environmental variables, but a general mechanistic basis of the response has been difficult to formulate [2]. This might be due to the fact that Fick's first law of diffusion does not account for the spatial distribution of the gas exchange in relation to microstructural features such as cell arrangement, size or cell wall thickness. Moreover, chloroplast movement in the cytoplasm, carbonic anhydrase (CA) activity in different cellular organelles and the amount and role of cooporins in the membranes may contribute in facilitating CO_2_ uptake [3], [20], [21], [22].

Correlations of 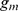 with leaf microstructural properties have not always been clear [2]. One reason is probably that mostly single structural properties were considered in these studies described by simple parameters, such as leaf porosity or leaf mass per area. However, leaf microstructure is a complex assembly of cells of varying sizes and with tortuous connections, interlaced with distorted intercellular spaces that will affect the actual diffusion pathway in the leaf. Insight in the relation between these microstructural features and photosynthesis requires a detailed model that incorporates the microstructural geometry of the leaf. Microscale exchange of CO_2_ in leaves has been investigated using theoretical models [23], [24]. In these studies, tissue models were constructed by means of basic geometrical elements such as spheres and cylinders. However, these models were relatively crude compared to the actual irregular microstructure of the tissue. Also, they did not take into account the exchange barriers of biological membranes which recently were shown to be important [25]. Tholen and Zhu [3] very recently developed a 3-D model for gas transport in a single generic C_3_ mesophyll cell. The model incorporated reaction diffusion equations for CO_2_ and 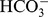 and included all cellular microstructural features of the CO_2_ transport pathway and associated reactions. However, being a model for CO_2_ transport within a single cell, it does not consider potential resistances within the intercellular space and, more importantly, any additional resistances due to cells being attached to each other and possibly reducing the exchange surface for CO_2_ considerably.

Recently, a mathematical microscale gas exchange model was developed to describe gas movements in fruit tissue through the intercellular space and cells by the authors [26], [27]. The gas exchange model was based on the actual microscale geometry of the fruit tissue and accounted for both gas diffusion as well as respiration kinetics. The model was used to evaluate the effect of ambient conditions, fruit size and maturity on the intracellular O_2_ and CO_2_ concentrations in fruit in relation to the occurrence of anaerobis via *in silico* analysis [27], [28]. In principle this model could also be used to describe microscale gas exchange in leaf tissue if the rate equations for leaf photosynthesis would be incorporated. The latter have been constructed by Farquhar, von Caemmerer and Berry [29] – the so-called FvCB model – which has been widely used for describing C_3_ photosynthesis. This biochemical model has also been coupled to a simple (lumped) CO_2_ exchange model [30], [31], [32], [33]. Yin et al. [17] have recently shown how to use combined measurements of gas exchange and chlorophyll fluorescence to estimate parameters of the FvCB model.

The objectives of this article were (i) to develop a microscale model for CO_2_ exchange through the leaf by coupling a detailed biophysical model of gas diffusion that incorporates the actual microstructure of the leaf to the biochemical FvCB model of photosynthesis; (ii) to validate the model with independent data, (iii) to quantify the importance of the different pathways of gas exchange; and (iv) to analyze the response of 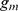 and 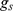 to environmental factors such as CO_2_ and irradiance. Wheat (*Triticum aestivum* L.) leaf was chosen as a model system.

## Results

### Microscopic gas concentration distribution

Mesophyll tissue contains a loose arrangement of cells in a large intercellular space. However, cells inevitably touch each other, thereby reducing the gas exchange surface area and introducing an additional, local resistance to CO_2_ transport. This would translate into local CO_2_ concentration gradients. We decided to carry out some simulations to test this hypothesis with a microscale model that combines a diffusion model for CO_2_ and 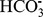 with the FvCB model for CO_2_ fixation in the chloroplasts and incorporates the actual 2-D leaf tissue microstructure.

**Figure 1:**
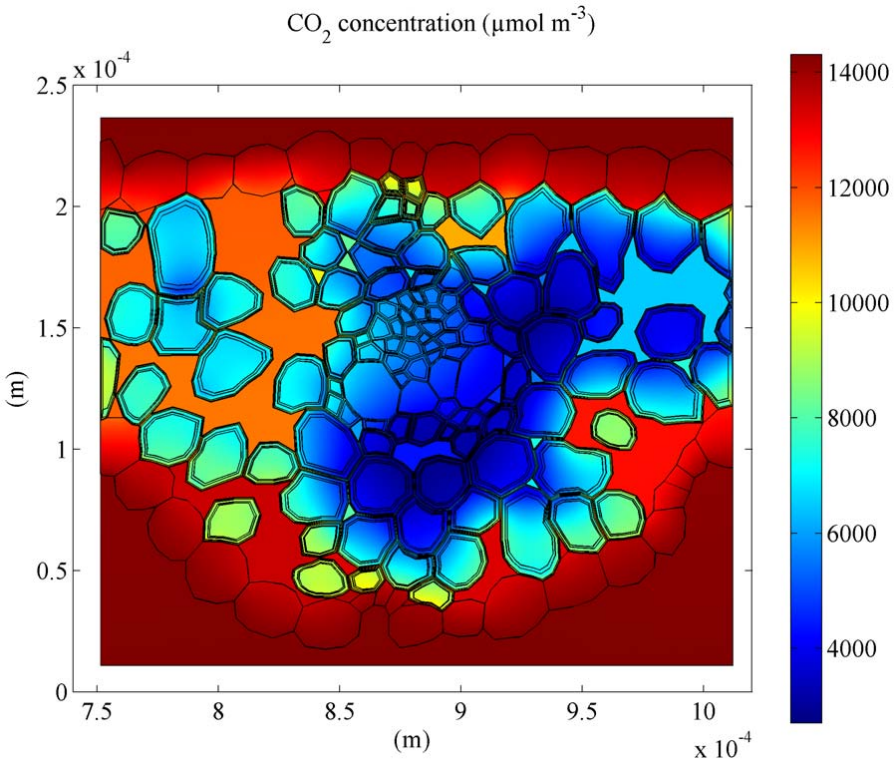
Computed CO_2_ distribution in wheat leaf. The ambient conditions were 350 µmol mol^−1^ CO_2_, 21% O_2_, 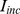 = 1000 µmol m^−2^ s^−1^ and 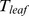 = 25°C. Concentrations are expressed in µmol m^−3^.

The CO_2_ distribution computed by the microscale model for the wheat leaf corresponding to ambient conditions of 350 µmol mol^−1^ CO_2_, 21% O_2_, 1000 µmol m^−2^ s^−1^ 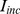 and 25°C is shown in Figure 1. The meaning and units of all symbols are given in Table 1. As expected, the CO_2_ concentration in the pores is considerably higher than inside the mesophyll cells. However, the concentration in the intercellular space is definitely not uniform, probably due to the relatively compact mesophyll tissue microstructure of wheat leaves compared to that of other species. Further, relatively large CO_2_ gradients can be observed within cell clusters. For this particular mesophyll tissue, the resistance to CO_2_ transport is clearly not negligible.

**Table 1:** List of model variables, their symbols and definitions.

Variable	Definition
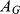	Gross photosynthesis rate (µmol CO_2_ m^−2^ s^−1^)
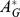	Gross volumetric photosynthesis rate of chloroplast (µmol CO_2_ m^−3^ s^−1^)
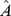	Measured net photosynthesis rate (µmol CO_2_ m^−2^ s^−1^)
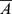	Mean net photosynthesis rate computed from microscale model (µmol CO_2_ m^−2^ s^−1^)
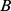	Net hydration of CO_2_ to 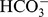 (mol m^−3^ s^−1^)
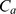	Ambient air CO_2_ concentration (µmol mol^−1^)
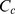	Mesophyll CO_2_ concentration (µmol mol^−1^)
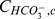	HCO_3_^−^ concentration of the mesophyll (mol m^−3^)
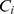	Intercellular CO_2_ concentration (µmol mol^−1^)
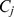	CO_2_ concentration in phase *j*
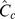	Measured mesophyll CO_2_ concentration using combined gas exchange and chlorophyll fluorescence measurements (µmol mol^−1^)
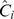	Measured intercellular CO_2_ concentration (µmol mol^−1^)
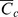	Mean mesophyll CO_2_ concentration computed from microscale model (µmol mol^−1^)
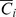	Mean intercellular CO_2_ concentration computed from microscale model (µmol mol^−1^)
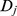	Diffusivity of phase *j* (m^2^ s^−1^)
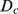	Diffusivity of CO_2_ in the mesophyll cytoplasm (m^2^ s^−1^)
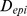	CO_2_ diffusivity of epidermis layer (m^2^ s^−1^)
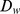	CO_2_ diffusivity of cell wall (m^2^ s^−1^)
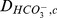	Diffusivity of HCO_3_^−^ in the mesophyll cytoplasm (m^2^ s^−1^)
*d*	Average thickness of tissue (m)
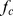	The fraction of chloroplasts of the leaf
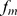	The fraction of cytosols of the leaf
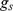	Stomatal conductance (mol m^−2^ s^−1^)
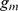	Mesophyll conductance (mol m^−2^ s^−1^)
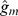	Measured mesophyll conductance using combined gas exchange and chlorophyll fluorescence measurements (mol m^−2^ s^−1^)
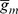	Computed mesophyll conductance from Eq. 14 (mol m^−2^ s^−1^)
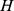	Henry's constant for CO_2_ (molm^−3^ liquid) (mol m^−3^ gas)^−1^
[*H^+^*]	H^+^ concentration (mol L^−1^)
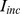	Photon flux density incident to leaves (µmol photon m^−2^ s^−1^)
*J*	Rate of potential electron transport calculated from chlorophyll fluorescence measurements (µmol electron m^−2^ s^−1^)
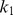	CO_2_ hydration velocity constant (s^−1^)
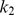	CO_2_ dehydration velocity constant (s^−1^)
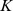	Acid dissociation constant for H_2_CO_3_ (mol L^−1^)
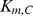	Michaelis-Menten constant of Rubisco for CO_2_ (µmol mol^−1^ or μbar)
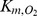	Michaelis-Menten constant of Rubisco for O_2_ (mbar)
*O_2_*	Oxygen partial pressure (mbar)
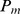	CO_2_ permeability of cell membrane (m s^−1^)
*R*	Universal gas constant (8.314 J mol^−1^ K^−1^)
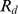	Day respiration (i.e. respiratory CO_2_ release other than by photorespiration) (µmol CO_2_ m^−2^ s^−1^)
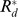	Volumetric respiration rate (µmol CO_2_ m^−3^ s^−1^)
*s*	Slope factor for converting chlorophyll fluorescence-based PSII electron efficiency into *J* (−)
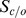	Relative CO_2_/O_2_ specificity factor for Rubisco (mbar μbar^−1^)
*T_leaf_*	Temperature of the leaf (K)
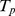	Rate of triose phosphate export from the chloroplast (µmol m^−2^ s^−1^)
*t*	Time (s)
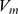	Total mesophyll cells volume (m^3^)
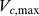	Maximum rate of Rubisco activity-limited carboxylation (µmol m^−2^ s^−1^)
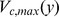	The relative photosynthetic capacity at a depth *y* inside the leaf
*w_c_*	Rate of Rubisco activity-limited carboxylation (µmol m^−2^ s^−1^)
*w_j_*	Rate of electron transport-limited carboxylation (µmol m^−2^ s^−1^)
*w_p_*	Rate of TPU-limited carboxylation (µmol m^−2^ s^−1^)
*w*(*y*)	The width of the leaf at the depth *y* (m)
*y*	The depth of the leaf from adaxial surface (m)
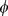	CO_2_ flux through the membrane (µmol m^−2^ s^−1^)
Γ*	*C*_c_-based CO_2_ compensation point in the absence of *R_d_* (µmol mol^−1^ or μbar)

The unit µmol mol^−1^ for CO_2_ concentration (often used in the FvCB model) was converted to µmol m^−3^ for use in the gas diffusion model by multiplying with a factor 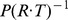 for CO_2_ concentration in the gas phase and 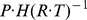 for CO_2_ concentration of the mesophyll, respectively. *P* (Pa) is the total pressure of the ambient air, *R* (J mol^−1^ K^−1^) is the universal gas constant and *T* (K) is the temperature.

**Figure 2:**
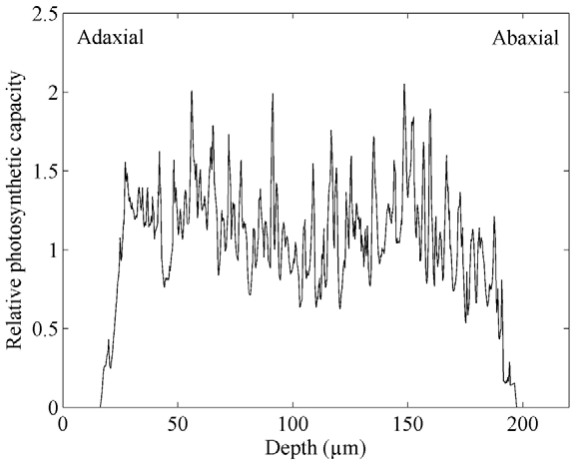
Distribution of the relative photosynthetic capacity along the depth of the wheat leaf computed from the modelled microscale geometry.

A detailed analysis of the calculated resistances of the different compartments of the leaf tissue is shown in Table 2. The resistance of the chloroplast envelope contributed up to 11.43% of the total resistance. This suggests that the chloroplast envelope effectively contributes significantly to the resistance to CO_2_ transport in the mesophyll cells, confirming the simulation results of Tholen and Zhu [3] for single mesophyll cells. Microscale simulations with a lumped intracellular compartment (without distinguishing the individual chloroplasts or other organelles) have been additionally carried out (Text S1, Figure S1). These results showed that there was a good similarity in total gas flux between the lumped model and the one with the chloroplasts taken into account the resistance of the chloroplast envelope; the latter, however, predicted a *g_m_* that was 12.7% higher than that obtained with the lumped intracellular model. Apparently, the reduced resistance to CO_2_ transport due to the position of the chloroplasts near the plasma membrane outweighs the increased resistance due to the double membrane of the chloroplasts compared to the lumped model. The modelled distribution of 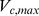 along the depth of a typical leaf is shown in Figure 2. There is a decreasing trend at the abaxial side of the leaf. Also, there is a dip where there is a vascular bundle.

**Table 2:** Resistance analysis of different compartments of the wheat leaf described in the model, for the CO_2_ diffusion from ambient air to chloroplast stroma.

	Resistance	
	(m^2^ s mol^−1^)	(%)
Epidermis	1.38	16.89
Intercellular space	2.54	31.10
Cell wall	1.89	23.05
Plasma membrane	0.44	5.37
Cytosol	0.52	6.38
Chloroplast envelope	0.94	11.43
Stroma	0.47	5.78
Total	8.18	100.00

The resistances were calculated by dividing the average concentration difference across compartments by the average flux expressed per unit of exposed leaf surface.

### Photosynthesis in response to CO_2_ concentration and model validation

In a next step, we investigated whether the microscale model was able to predict the measured response of leaf photosynthesis to the ambient CO_2_ concentration in photorespiration conditions. The following convention for symbols is used further: macroscopic variables which were estimated from gas exchange and chlorophyll fluorescence experiments are denoted by a ‘∧’ symbol. Volume averaged variables calculated from the microscale model are overlined (see more details in Materials and Method section).

**Figure 3:**
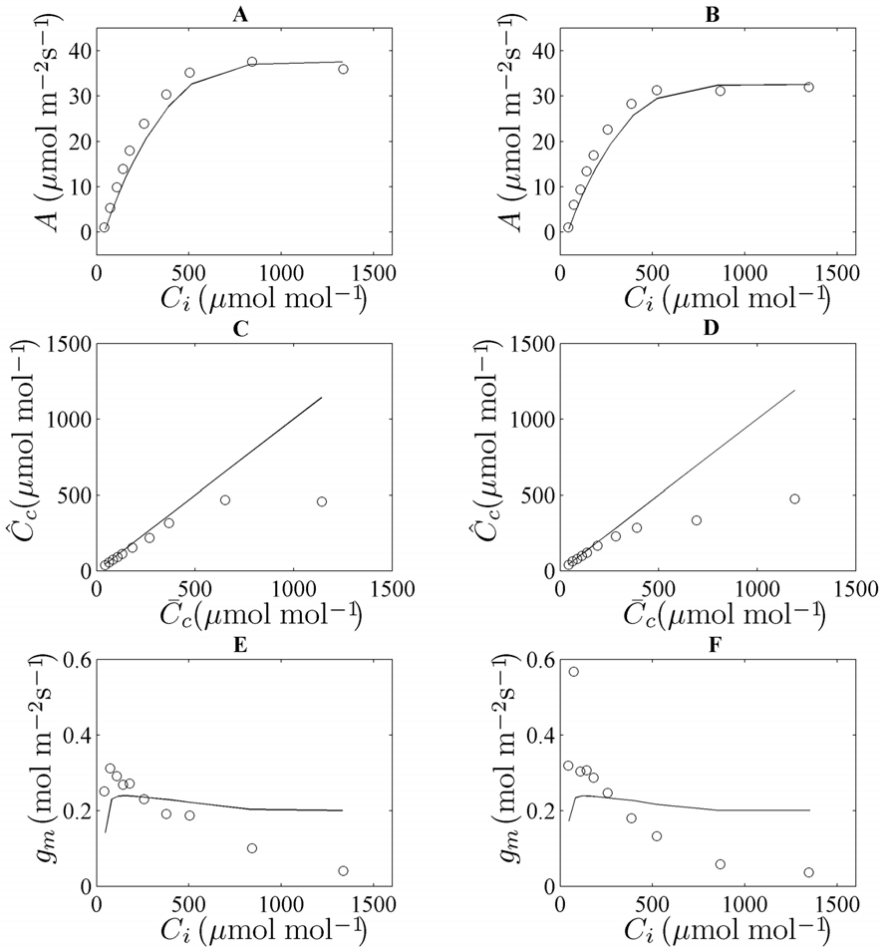
Simulations and measurements at different conditions of 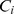 at 21% O_2_, 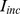 = 1000 µmol m^−2^ s^−1^ and 25°C at flowering stage. Figures (A) and (B) show *A* as function of 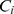 for the flag leaves at high and low N supply, respectively. The symbols represent measurements (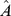 versus 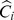) while the lines indicate model predictions (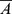 versus 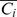). Figures (C) and (D) depict 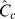 versus 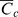 for high and low N supply flag leaves, respectively. The diagonal lines indicate perfect correspondence. Figures (E) and (F) show 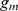 as function of 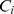 for high and low N supply flag leaves, respectively. The solid (—) line represents 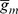 versus 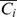. The symbols (o) represent the measured data (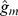 versus 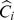). Data are from Yin et al.[17].

Plots of the measured and simulated net photosynthesis rate at 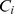 values from 50 to 1500 µmol mol^−1^ at 1000 µmol m^−2^ s^−1^ 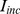 and 21% O_2_ are shown in Figure 3. A good agreement was found between measured and simulated data. 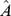 rapidly increased at low 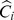 concentrations but saturated at high CO_2_ concentrations (Figures 3A&3B). The relationship between 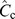 and 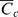 is shown in Figures 3C&3D. They are approximately equal at low CO_2_ concentrations (<500 µmol mol^−1^), but at high CO_2_ concentrations 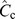 levels off. In Figures 3E & 3F, 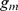 is plotted as a function of 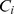. Excluding the low-CO_2_ region where any assessment of *g_m_* is uncertain [1], [2], clearly 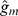 decreased with increasing CO_2_ levels; 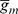 also decreased with increasing CO_2_ levels but then stabilized at high CO_2_ concentrations. Similar results were found when validating the model using data obtained from wheat leaves at 2 weeks after flowering (Figure S2).

**Figure 4:**
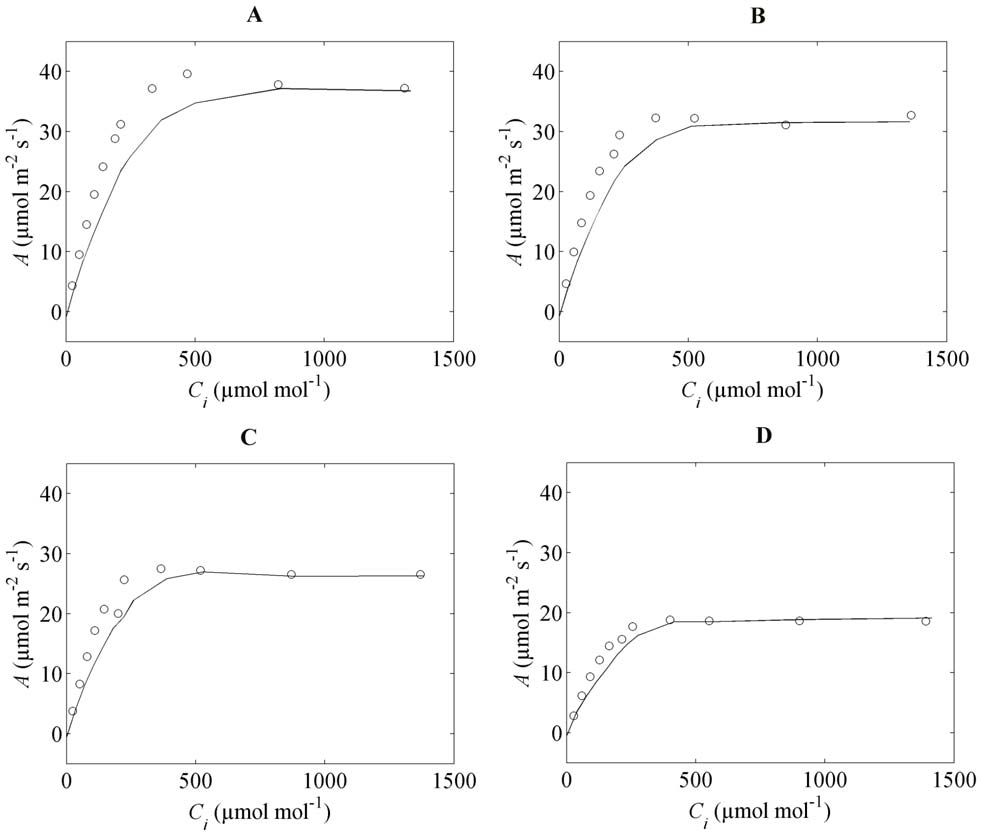
CO_2_ response of net CO_2_ assimilation rates of the flag leaves under the conditions of 2% O_2_. (A) and (B) correspond to flag leaves at high N and low N supply at flowering while (C) and (D)correspond to flag leaves at high N and low N supply at two weeks after flowering. The symbols represent the measured values of  versus 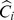 [17]; the solid (—) represent the computed 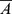 versus 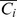.

We then validated the microscale model using data obtained at 2% O_2_. The computed CO_2_ assimilation rate was slightly underestimated compared to the measurements (Figure 4), especially for the condition of high and low N supply at flowering stage (Figures 4A&4B).

### Photosynthesis in response to irradiance

**Figure 5:**
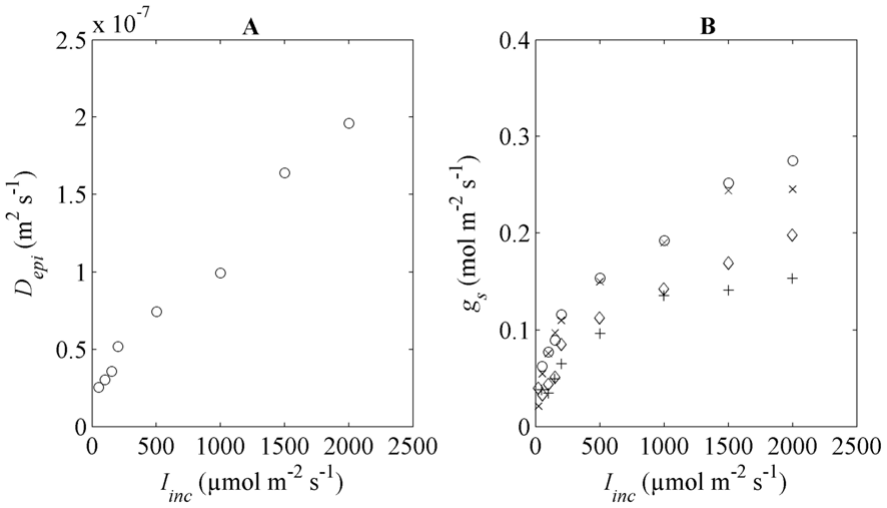
Epidermal diffusion and CO_2_ stomatal conductance as function of 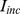. (A) Fitted epidermal diffusion (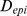) as function of 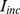. (B) Measured CO_2_ stomatal conductance (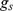) as a function of 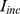. The symbols (o) and (×) represent high and low N supply flag leaves at flowering stage, respectively while symbols (◊) and (+) represent high and low N supply flag leaves at two weeks after flowering.

Yin et al. [17] found that 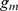 and 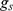 increase with increasing 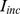. We wanted to evaluate whether the microscale model indeed predicts such behaviour. Microscale gas exchange simulations were carried out for different values of 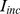 increasing from 0 to 2000 µmol m^−2^ s^−1^ (350 µmol mol^−1^ 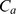 and 21% O_2_). If using a constant 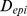 = 1.67×10^−7^ m^2^ s^−1^ (Table 3), the CO_2_ concentration in the intercellular space was overestimated by the model for the conditions of low light intensity (results not shown). As 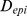 was considered in the microscale model as a lumped parameter that included the gas diffusion through the stomata, its value was expected to vary with irradiance. The high N data at flowering stage were used for fitting 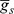 to 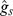 and to determine 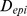. The effects of light on 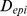 and 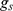 are shown in Figure 5. The results confirm that 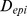 and 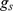 increase with 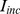, due to the opening of the stomata by light [34].

**Table 3:** Physical parameters of the microscale gas exchange model.

Model parameters	Symbol	Values
Diffusivity		
- Pore	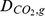	1.60×10^−5^ m^2^ s^−1^ at 20°C(a)
- Cytosol and stroma	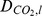	1.67×10^−9^ m^2^ s^−1^ at 20°C(a)
- Cell wall	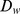	3.437×10^−7^ m^2^ s^−1^
- Epidermis	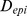	1.672×10^−7^ m^2^ s^−1^
	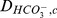	1.17×10^−9^ m^2^ s^−1^ (b)
Cell wall thickness	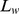	0.5 µm
Membrane permeability	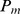	3.5×10^−3^ m s^−1^ (c)
Henry's constant	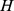	0.83 (mol m^−3^ liquid)(mol m^−3^ gas)^−1^ at 25°C(a)
CO_2_ reaction rate constants	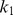	0.039 s^−1^ (d)
	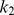	23 s^−1^ (d)
	*K*	2.5×10^−4^ mol L^−1^ (d)

(a) Lide [43],

(b) Geers and Gros [76],

(c) Gutknecht et al. [47],

(d) Jolly [77].

Symbols are defined in the Table 1.

**Figure 6:**
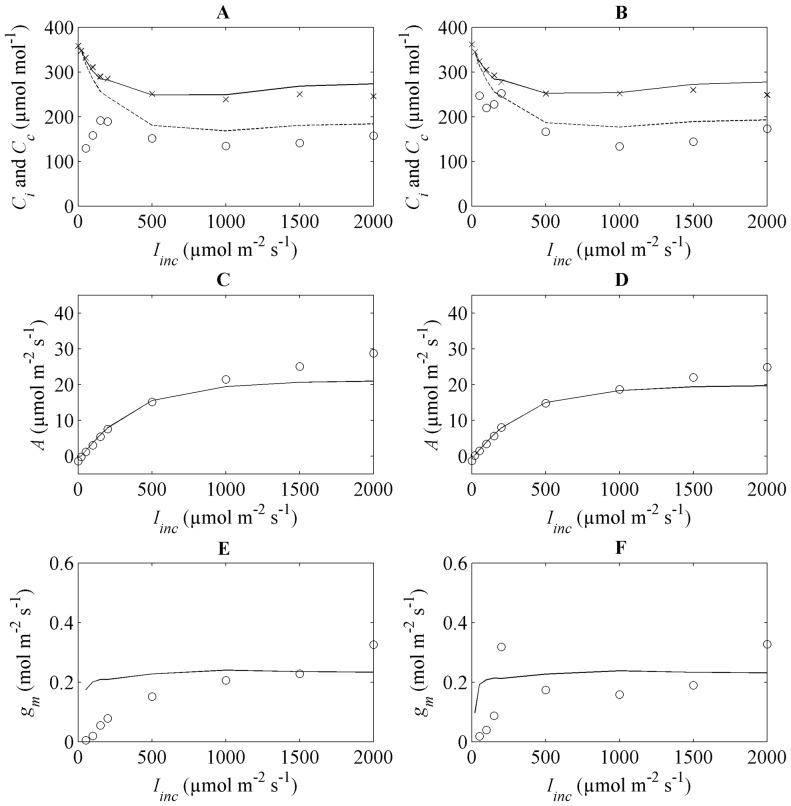
Model predictions (lines) versus measurements (symbols) of photosynthesis variables for 350 µmol mol^−1^ CO_2_, 21% O_2_, 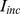 from 0 to 2000 µmol m^−2^ s^−1^ and 25°C at flowering stage. Left figures represent fitting results using data from high N supply flag leaves; right figures were simulations for low N supply flag leaves. Figure (A) and (B) show 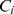 and 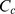 as a function of 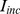; solid lines (—) and dashed lines (- -) represent 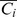 and 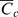, symbols (×) and (o) represent 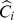 and 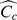, respectively. Figure (C) and (D): *A* as function of 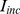. Figure (E) and (F): mesophyll conductance 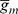 (—) or 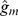 (o) as function of 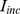. Data from Yin et al. [17].

The 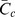 values were larger than the measured ones at low 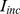 while at high values of 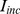 both *C_i_* and *C_c_* in the model and measurement levelled off (Figures 6A&6B). 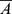 as a function of 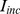 agreed well with the measured values at low 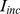 but was underestimated at high 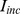 (Figures 6C&6D). While 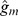 seemed to be very sensitive at low 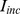, 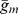 was not (Figures 6E&6F). Similar results were found for validation on wheat leaf at 2 weeks after flowering (Figure S3). Overestimations of 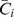 and 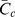 compared to the measurements were found. Note that the 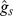 obtained for two weeks after flowering was lower than the 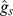 at the flowering stage, while the values of 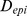 at different 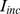 applied in the simulation resulted in 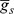 similar to 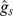 for the high N leaves at flowering stage.

### Microstructure effect on mesophyll conductance

**Figure 7:**
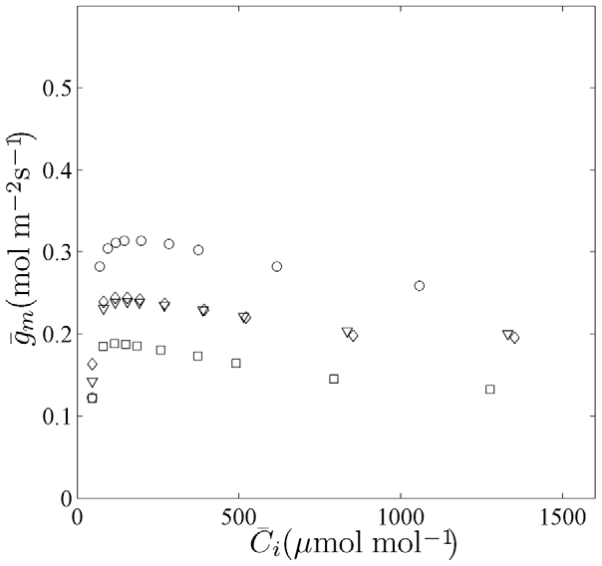
Model predictions of 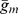 as a function of 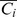 in high N supply flag leaves at flowering stage using four different microstructure topologies of wheat leaves. The simulations were done for different external CO_2_ concentrations from 50 to 1500 µmol mol^−1^, 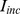 = 1000 µmol m^−2^ s^−1^ in photorespiration conditions (21% O_2_). Different symbols correspond to different microstructure topologies.

The anatomy of the leaf may have an effect on microscale gas exchange and result in variation in mesophyll conductance. In order to test this hypothesis, the mesophyll conductance was computed for four different micro-structures of a wheat leaf based on light microscopic images at 15, 30, 60 and 90 mm above the leaf base taken from the literature [35]. Simulations were carried out at different values of 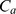 from 50 to 1500 µmol mol^−1^,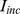 = 1000 µmol m^−2^ s^−1^ in photorespiration conditions (21% O_2_). In Figure 7 the computed values of 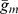 for four different microscale geometries are shown as a function of 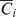. The 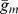 values varied for the different microstructures, validating our hypothesis. A decreasing trend of 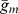 with increasing 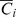 was found consistently, irrespective of leaf microstructures. This is a simulation result that follows from the model and it is difficult to trace this to a particular submodel.

## Discussion

### CO_2_ transport model

Fick's diffusion equation is applicable to transport of a chemical species such as CO_2_ in a continuum material such as water. It can be related to Brownian motion according to the Einstein–Smoluchowski equation that has its foundations in statistical mechanics. Several authors have used the diffusion equation to describe CO_2_ uptake by leaves [36]. Such models were solved with geometrical simplifications such as a 1D model of CO_2_ drawdown in the leaf [37], a restricted and simplified zone analysis of diffusion from a small sub-stomatal cavity into a hemispherical region surrounding it [38], and CO_2_ diffusion through a single stoma and the surrounding mesophyll using an axial symmetry model [23]. Aalto and Juurola [24] constructed a 3-D model for CO_2_ gas exchange through the leaf with basic geometrical elements such as spheres and cylinders representing mesophyll cells. While in their model the cells were separated by air gaps, in reality cells touch each other and this contact may reduce both the surface available for CO_2_ exchange and the diffusion among the cells as we have clearly shown. The most realistic photosynthesis model to date was recently described by Tholen and Zhu [3]. Their model, while addressing 3-D CO_2_ transport in a single mesophyll cell and incorporating subcellular features such as chloroplasts and mitochondria, does not account for any resistances due to the leaf microstructure and in particular the mesophyll.

In our model we incorporated for the first time the actual microstructure as observed from microscopy images in the CO_2_ transport model. We considered six materials (epidermis, cell wall, cytoplasm, chloroplast, vacuole and air) and we assumed that these materials were proper continuum materials so that we could assume Fickean diffusion of CO_2_ within each of them. Membranes were modelled as resistances. In contrast to the model of Aalto and Juurola [24], our model does account for the effect of mesophyll cells touching each other and thereby reducing the exchange surface between mesophyll and intercellular space. Further, our simulations show that wheat leaves with different microstructure have widely different 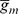 values (Figure 7), indicating a clear effect of microstructure on gas transport (also see next section). This implies that our model is in principle not restricted to leaf types in which air space resistance is negligible as in the model of Tholen and Zhu [3].

We carried out a simulation in which we replaced air by helox in the model, corresponding to an increase of the diffusivity of CO_2_ in the gas phase by 2.33 compared to that of the original model. At ambient conditions of 350 µmol mol^−1^ CO_2_, 21% O_2_, 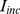 = 1000 µmol m^−2^ s^−1^ and 25°C, *A* was 6.8% higher than in the case of the air. This corresponds to the results of Parkhurst and Mott [38] who experimentally found that *A* was up to 7% higher in the amphistomatous leaves compared to air and up to 27% higher for the hypostomatous ones. While we did not do any measurements with helox, this result provides additional evidence that our model predicts realistic results. Additionally, it indicates that the intercellular space affects CO_2_ transport and thus photosynthesis. Note that a lumped model, in contrast, cannot explain the effect of helox on photosynthesis

The effect of nitrogen treatment on the photosynthetic parameters of wheat leaves at different development stages was investigated by Yin et al. [17]. A relatively small effect of nitrogen treatment could be observed in the flowering stage; two weeks after flowering the effect was somewhat larger (Figures 3, 6; Figure S2 and S3). The effect of development stage was, however, considerable (Figures 3, 6; Figure S2 and S3). The more significant difference in the later stage was probably due to the greater difference in the content of leaf nitrogen as large amount of leaf nitrogen was translocated into grains during grain filling.

We calibrated and validated the model at one temperature (25°C), as data were available for this temperature only [17]. However, temperature is known to have a large effect on photosynthesis [39], [40], [41], [42]. The temperature dependence of physical constants such as the solubility and diffusivity of CO_2_ and 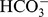 is known [44]. Also, mathematical expressions have been developed to describe the temperature dependence of the parameters of the FvCB model for different species [39], [40], [41], but not for wheat. In fact, the values of the activation energy of *V_c,max_* and *J_max_* used by De Pury and Farquhar [43] and Archontoulis et al. [43] for wheat were actually obtained by Badger and Collatz [44] from experiments with *Atriplex glabriuscular* leaf and by Farquhar et al. [29]. Preliminary simulations with temperature dependent *V_c,max_* and *J_max_* values taken from these references showed that the net photosynthesis of wheat leaves is highly dependent on temperature (Figure S4). Additional experiments are required to determine the temperature dependence of the parameters of the photosynthesis kinetics of wheat.

In our model it is assumed that CO_2_ transport in the cell occurs mainly in the form of CO_2_ and HCO_3_^−^ depending on the local pH. The dissociation of HCO_3_^−^ to H^+^ and 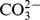 is not significant at pH values below 8. There is both theoretical and experimental evidence for significant carbonic anhydrase (CA) dependent facilitation of CO_2_ transport in C_3_ plants [20], [22], [45]. CA isozymes may be active in different cellular components [22], [46] and may affect CO_2_ transport. In fact, Tholen and Zhu [3] calculated that removing all CA from the stroma would reduce *g_m_* by 44%. As little information is available about the rate constants of the hydration and dehydration of CO_2_ by CA, or its activity in the different organelles of the cell, we decided at this stage to not include CA activity in the microscale model until more information would become available; incorporation in the model would be straightforward and desirable, though.

The value of *P_m_* was taken from Evans et al. [20] and Tholen and Zhu [3], who used the results of Gutknecht et al. [47] from experiments with equimolar mixtures of egg lecithin and cholesterol. The chemical composition of such a bilayer is, however, likely to be different from that of the cellular membranes of wheat leaf. The permeability of both the plasma and chloroplast membrane has also been shown to depend on the amount of embedded aquaporins (cooporins) [25]. In fact, Evans et al. [20] found values for *P_m_* ranging from 10^−6^ to 1.6×10^−2^ m s^−1^ in the literature. When we used the value reported by Uehlein et al. [25] (*P_m_* = 0.8×10^−6^ m s^−1^) we obtained a value of 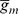 that was considerably smaller than the measured one. More research on cell membrane permeability of plants and wheat in particular is thus required.

The microscale model described here does not consider the light profile inside the leaf yet. Coupling a full light penetration model to this model may be very helpful to estimate the distribution of quanta that are absorbed by the mesophyll cells within the leaf for photosynthesis. Future research thus should also address models for light propagation in leaf tissue.

### Effect of leaf microstructure on CO_2_ diffusion

During photosynthesis, CO_2_ moves from the atmosphere surrounding the leaf to the sub-stomatal internal cavities through stomata, and from there to the site of carboxylation inside the mesophyll cells. The simulation results indicated that gas exchange through the microstructure is very heterogeneous. Large gradients and low CO_2_ concentrations were mainly found inside the mesophyll cells and cell clusters due to photosynthesis and limited diffusion of CO_2_ in the mesophyll cells. The CO_2_ concentration at the carboxylation site in the chloroplast stroma, *C_c_*, in C_3_ plants is lower than *C_i_* [3], [11], [48], [49]. The diffusion barriers such as the water-filled pores of the cell wall, plasma membrane, cytosol, the envelope and stroma are responsible for the resistance of CO_2_ along the pathway from intercellular space to stroma [20]. Several authors (Evans and von Caemmerer [11], Evans et al. [14], Evans et al. [20], Terashima et al. [49]) reported that chloroplasts adhere exclusively to the plasmamembrane of mesophyll cells and, therefore, path length of CO_2_ transport over the cytoplasm is reduced. Tholen et al. [21] indicated the possibility of chloroplast movement that may have significant consequences for the diffusion of CO_2_ through the mesophyll. Simulations with a microscale model with chloroplasts lumped over the mesophyll cells showed that the predicted value of *g_m_* was lower than when they incorporated chloroplasts near to the cell wall. This indicates that the position of the chloroplasts next to the plasma membrane does indeed reduce the resistance for CO_2_ transport.

The distribution of 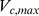 depends on the distribution of chlorophyll through the leaf and the presence of the vascular region. In *Eucalyptus pauciflora* leaves, the photosynthesis capacity has been shown to be low in the vascular bundle region [50]. Evans and Vogelmann [51] showed that with increasing depth the photosynthetic capacity first increased followed by a strong decrease which finally levelled off in spinach leaves. This was not implemented in our model as there was no data available for wheat.

Early literature has assumed that simple diffusion through cellular membranes [52] and/or leaf structural features [14], [53], [54] are responsible for most of the variation in 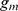. Flexas et al. [2] supposed that 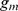 can be correlated to some leaf microstructural features. Our simulation results provided even more direct evidence of gas concentration gradients in relation to the microstructure topology of leaves and the effect of variation of the leaf microstructure on 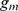: depending on the value of 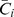 , the value of 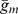 that was computed for different microstructure topologies was 30% different from the mean value (Figure 7). Biological variation thus considerably affects the mesophyll conductance. This may depend on the species, though: the microstructure of wheat leaf mesophyll is relatively tight compared to that of other species. Future photosynthesis models should thus not simply ignore the tissue microstructure.

The epidermis was implemented as a homogeneous layer without explicitly modelling the stomata, resulting in a high value of 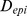. The positive dependence of 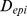 on 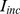 (Fig. 6) is most probably due to the aperture of the stomata in response to the light. The cell walls were modelled as channels connecting the larger pores in the tissue, thereby creating a void network structure that facilitates gas exchange resulting in a high diffusivity of cell wall (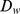). When the cell wall structure was assumed to be saturated with liquid in the 2D model, the net CO_2_ assimilation flux decreased drastically compared to the measurement and resulted in a significant underestimation of mesophyll CO_2_ concentration. Evans et al. [20] showed that CO_2_ diffusivity of the cell wall (1.7×10^−9^ m^−2^ s^−1^) was much smaller than the value obtained here (see Table 3). As *in vivo* the cell walls are expected to be fully hydrated, this may indicate that the interconnectivity of the microstructure is considerably larger than expected from the 2-D microscale geometry. Consequently, *D_w_* is in our model an apparent parameter that accounts for both CO_2_ diffusion in the cell wall but also for the connectivity of the intercellular space in 3-D. Lateral gas diffusion within the intercellular air space has been studied by Pieruschka et al. [55] and Morison et al. [56]. Morison et al. [57] indicated that the supply of CO_2_ from nearby stomata usually dominates assimilation, but that lateral supply over small distances can be important if stomata are blocked, particularly when the assimilation rate is low. The discrete positions of stomata may thus have an influence on the diffusion gradients in the leaf. As the 2-D model described here cannot fully capture gas transport through and from discrete stomata, a 3-D microscale gas transport simulation in a real leaf geometry is required to understand lateral gas diffusion in the leaves. A 3-D network structure with strong connectivity has indeed been observed in several plant tissues such as fruits [58], [59], [60]. The 3-D microstructure of stomatal aperture and the corresponding microscale gas exchange through the stomata have recently been investigated using a diffusional resistance model [61]. Indeed, the 2-D gas exchange model described here is an important step toward a realistic full 3-D gas exchange model based on 3-D microstructure of leaf tissue which has not been achieved so far. The extension of our model to a 3-D model requires the geometrical model to be changed from 2-D to 3-D which is not trivial and requires advanced 3-D visualisation techniques such as synchrotron X-ray micro computed tomography [60]. The model equations, however, do not need to be changed.

It is important to note that our microstructural model (and a possible 3-D extension) complements rather than replaces the lumped approach for photosynthesis modelling that has been used by many authors [1], [5], [10], [11], [12]. A lumped model, even when it fits GE/CF measurements very well, does not improve our understanding on the role of mesophyll porosity, cell size, presence of vascular bundle or any other microstructural features on photosynthesis. Our 2-D model (and a future 3-D even more) does provide such information.

### Effect of CO_2_ and irradiance on mesophyll conductance

We confronted our model extensively with measured gas exchange and chlorophyll fluorescence data and obtained in general a good agreement between simulated and measured values. However, the model failed to predict the decrease of 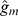 at high CO_2_ values that was seen in the measurements and that is a topic of current debate [1], [17].

One explanation for this mismatch could be the uncertainty on the estimation of 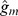 based on combined gas exchange and chlorophyll fluorescence measurements, and the estimation of Harley et al. [10], Yin and Struik [12]. The latter authors found that the estimated mesophyll conductance becomes increasingly sensitive to variations of the measurements as the value of 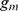 increases, and can be affected by both statistical artifacts in curve fitting and biological uncertainties in thylakoid stoichiometry [12]. In addition, Evans [62] and Terashima et al. [63] indicated that electron transport rates calculated from chlorophyll fluorescence may have potential errors, which the calibration procedure based on Equation (12) may not account for sufficiently. This would also explain the mismatch between 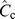 and 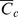 as observed in Figures 3C and 3D. However, the large discrepancy between 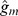 and 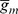 appears already at intermediate levels of *C_i_*, and is thus not well explained by these considerations. Another, more plausible, explanation may be that there are effects that have not been incorporated in our model. For example, Tholen and Zhu [3] used a gas transport model for single mesophyll cells to show that increasing the permeability of the chloroplast membrane for 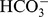 would indeed explain decrease of 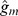 as a function of *C_i_*. Also, transport through the chloroplast membrane may be regulated by CA: CO_2_ diffuses more easily through membranes than HCO_3_^−^, so any regulatory mechanism that would affect the expression of CA and thus the equilibrium between CO_2_ and HCO_3_^−^ in different cellular compartments would also affect their transport through the relevant membranes. Finally, cooporins have been shown to be present in chloroplast membranes and may significantly affect membrane permeability [25]. These mechanisms may also explain the discrepancy between 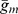 and 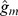 at low 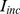.

## Materials and Methods

### Model assumptions

The following assumptions were made:

#### Model dimension

Gas transport is essentially 3-D. We have shown previously [60], [64] that in dense tissue such as in the cortex of fruit, pores that appear unconnected in 2-D may in fact be connected when visualised using 3-D techniques such as X-ray microfocus computed tomography (μCT). The reason that we have implemented a 2-D here instead of a 3-D model is the fact that μCT – the only feasible technique for 3-D visualisation of plant tissue at this resolution – provides insufficient contrast to discriminate organelles in a cell, and, for example, locate the position of the chloroplasts to include them in the geometrical model. Moreover, the best resolution that currently can be obtained with μCT (about 500 nm) is not enough to visualise the cell wall with sufficient contrast to allow segmentation of individual cells. This is a prerequisite for the method we used to artificially position the chloroplast layer inside the cell close to the plasmalemma (see further). As mesophyll is much less dense we expect that the difference between 2-D and 3-D is not as large as in fruit cortex tissue, but this remains to be investigated in future research.

#### Intercellular space

In contrast to the model of Tholen and Zhu [3], our model explicitly incorporated the actual microstructure of the mesophyll tissue, including the intercellular space and cells touching each other. This allows investigating any resistances these features may cause in addition to those investigated by the latter authors.

#### Cell organelles

Chloroplasts and mitochondria were modelled as different homogeneous layers in the cell rather than as individual organelles. This considerably reduced the complexity of the model and the required mesh density. This assumption was supported by the model of Tholen and Zhu [3] that displayed almost one dimensional gas exchange in a single isolated mesophyll cell one. It was further assumed that a mesophyll cell contained a single, large vacuole.

#### Stomata

In a 2-D model the real stomata distribution cannot be implemented without considerably overestimating the overall stomatal gas exchange of the leaf; only a true 3-D model would allow incorporating the stomata as such. We therefore modelled the epidermis layer as a continuum material with an effective diffusivity 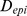. This lumped parameter implicitly incorporates stomatal gas exchange in such a way that the overall conductance of the epidermis in the model would be equal to the measured one.

#### Localisation of photosynthesis

We assumed that there was no photosynthesis in the epidermis and vascular bundle. Respiration was assumed to take place in the epidermis, the cytoplasm of mesophyll cells and phloem; xylem cells were assumed not to respire. Xylem was identified as large cells in the vascular bundle facing the adaxial epidermis.

#### Spatial dependence of photosynthesis rate

Several authors have found a spatial dependence of the photosynthesis rate [51], [65], [66]. The rate of photosynthesis across a leaf is determined by the light absorption profile and the profile of the photosynthetic capacity. With increasing depth the photosynthetic capacity first increases followed by a strong decrease and finally levels off. Although we realise that this would affect the modelling results, we did not find sufficient quantitative data on the spatial dependence of the photosynthesis rate in wheat.

#### Light transport

As light penetrates the leaf it is absorbed by the photosynthetic pigments and scattered at air-water interfaces. Palisade cells facilitate the penetration of collimated light into the inner parts of the leaf, whereas the spongy mesophyll scatters the light thus increasing the probability of the light being absorbed. Because of the difficulty of modelling of this process (for example by means of Monte Carlo methods) we have assumed here that the photon flux density is uniform in the leaf.

### Model of photosynthesis kinetics

The FvCB model was used in this article to describe the gross CO_2_ fixation rate *A_G_* in the chloroplasts of C_3_ plants [16], [29], [67], [68]. Briefly,
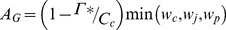
(1)with *w*_c_ the Rubisco-limited carboxylation rate, *w*_j_ the RuBP-regeneration or electron transport limited rate, and *w*_p_ the triose phosphate utilization (TPU) limited rate. They were calculated from
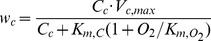
(2)

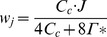
(3)

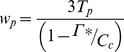
(4)with *C_c_* and *O_2_* the CO_2_ and O_2_ concentration in the chloroplast, respectively; *J* the rate of electron transport; *T_p_* the rate of triose phosphate export from the chloroplast; and 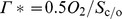 [17]. 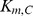, 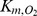 and 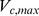 are constants. The meaning and units of all symbols are given in Table 1. The net photosynthesis rate *A* was defined as *A = A_G_-R_d_*, with *R_d_* the respiratory CO_2_ release other than by photorespiration.

### Microscale gas exchange model

The exchange of CO_2_ in the tissue was described by means of a reaction diffusion equation:
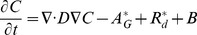
(5)

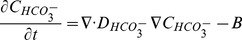
(6)with 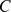 and 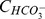 the local CO_2_ and 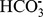 concentration; 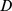 and 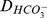 the corresponding local diffusivity coefficients; and *t* time. The volumetric photosynthesis rate 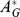 was assumed to be equal to zero everywhere except in the chloroplasts. 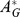 and 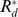 were calculated from 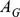 and 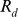 using
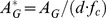
(7)

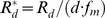
(8)with *d* (184 µm) the average thickness of the leaf, and *f_c_* (0.104) and *f_m_* (0.169) the fraction of chloroplasts and cytosol in a 2-D cross section of the leaf, respectively. *B* represents the net hydration rate of CO_2_ to HCO_3_^−^:
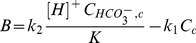
(9)The CO_2_ flux 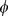 through the membranes of the cell, chloroplast and vacuole membranes was described by a flux boundary condition:
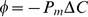
(10)with *P_m_* the membrane permeability that is equal to the reciprocal of resistance. It was assumed that the local CO_2_ concentration in the gas and liquid phase was always in equilibrium and described by Henry's law.

### Geometrical model

**Figure 8:**
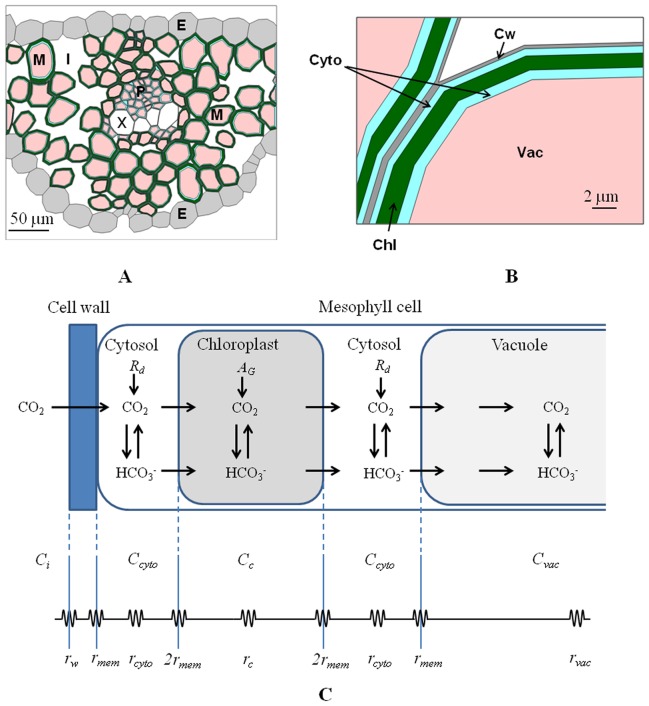
Reconstructed microscale geometry based on microscopic images of wheat leaf tissue and scheme of fluxes of CO_2_ species through different compartments of the mesophyll cell. (A) Reconstructed microscale geometry based on microscopic images of wheat leaf tissue [35]. The adaxial surface is at the bottom. E, epidermis; I, intercellular space; M, mesophyll cell; P, phloem; and X, xylem. (B) Detail of reconstructed mesophyll cells in computer model. Chl, chloroplast layer; Cyto, cytoplasm; Cw, cell wall; Vac, vacuole.(C) Scheme of fluxes of CO_2_ species through different compartments of the mesophyll cell and corresponding resistances. The resistances due to the epidermis, stomata and intercellular space are not included in this scheme. The symbols *C* and *r* indicate CO_2_concentration and resistance, respectively. The subscripts *i*, *w*, *cyto*, *c*, *vac* and *mem* indicate intercellular space, cell wall, cytoplasm, chloroplast, vacuole and membrane, respectively. The resistance of double membrane- chloroplast envelope was modeled as twice the resistance of the phospholipid membrane. *A_G_* is the gross photosynthesis rate; *R_d_* is respiration.

The 2-D geometry of wheat leaf was constructed from light microscopic images of wheat leaf available from the literature [35], as the experimental dataset of Yin et al. [17] did not contain microscopic images. As the leaf cross section consists of several similar parallel vein segments, only one segment was modelled and impermeable boundary conditions were applied at the left and right hand side of the geometrical model. The images were digitized in the Matlab programming environment version 7.0 (The Mathworks, Natick, MA) by in-house developed software (Figure 8). The cells were represented by polygons. The bottom and top cell layers constituted the epidermis. The thickness of plant cell walls generally lies in the range of 0.1 to 0.3 µm, but can exceed 1 µm [69], [70]. As it was not possible to determine the cell wall thickness accurately from the light microscopic images, we constructed the cell wall by shrinking the original polygon representing a cell by 0.5 µm normal to every edge; the volume between the original and shrunk polygon was defined as the cell wall. Since the model was solved using the finite element method, reducing the cell wall thickness would decrease the mesh size in the cell wall material and, hence, increase the required computational resources and time. This would not affect the model predictions appreciably as the cell wall thickness is interchangeable with *D_w_*: if we would have implemented a smaller cell wall thickness the parameter estimation procedure would have resulted in a larger value of *D_w_*, but the simulation results would be virtually identical. Chloroplasts appear as flat discs usually 2 to 10 µm in diameter and 1 µm thick. A mesophyll cell can contain 10 to 100 chloroplasts [71]. James et al. [72] found that the volume fraction of chloroplasts in the mesophyll cells was about 24%. For simplicity, chloroplasts were modeled as a layer located at a distance of 0.5 µm from the cell wall and occupying 20% of the modelled mesophyll cell volume. The relative photosynthetic capacity 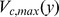 at a well defined depth *y* inside the leaf was calculated as
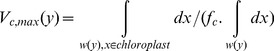
(11)where the integration is over the width *w*(*y*) of the leaf at the depth *y*. The distribution of photosynthesis capacity 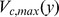 along the depth of the leaf depends on distribution of chlorophyll through the leaf, the presence of vascular region (Figure 2). The vacuolar volume fraction is variable and can be larger than 30% of the cell volume and up to 90% of the cell volume in a mature cell [71]. The vacuoles were modelled explicitly in the mesophyll cells by shrinking the cell area of 2D geometry by 60% and considering the shrunk area to be vacuole. For a spherical cell, for example, this corresponds to a vacuolar volume fraction of 46%. The layer between the cell membrane and the chlorophyll layer and that between the tonoplast and the chlorophyll layer was considered to be cytoplasm. This implies that CO_2_ to reach the vacuole has to pass the cell wall, the plasmalemma, twice the chloroplast membrane, and finally the tonoplast. In reality CO_2_ can diffuse directly from the plasmalemma to the tonoplast, but we believe that ignoring this only marginally affects intercellular CO_2_ transport while it simplifies the geometrical model considerably.

The resulting geometry of the tissue was then exported into a finite element simulation code (Comsol 3.5, Comsol AB, Stockholm, Sweden) via a Matlab interface. The leaf geometry and the corresponding finite element mesh that was used for the simulations are shown in Figure 8.

### Gas exchange and chlorophyll fluorescence measurements

Data used for our analysis came from measurements reported by Yin et al. [17] for photosynthesis of wheat plants grown under two contrasting levels of nitrogen supply. Nutrient supply is known to enhance photosynthesis, whereas it has a rather small and inconsistent effect on *g_m_* [73]. Simultaneous gas exchange and chlorophyll fluorescence measurements at both 21% and 2% O_2_ were performed on main-stem flag leaves at the flowering stage and two weeks after flowering, with four replications at each stage, using an open gas exchange system (Li-Cor 6400; Li-Cor Inc, Lincoln, NE, USA) and an integrated fluorescence chamber head (LI-6400-40; Li-Cor Inc, Lincoln, NE, USA). All measurements were made at a leaf temperature (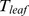) of 25°C and a leaf-to-air vapour pressure difference of 1.0–1.6 kPa. For the 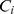 response curves, the ambient air CO_2_ concentration (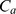) was increased step-wise: 50, 100, 150, 200, 250, 350, 500, 650, 1000, and 1500 µmol mol^−1^, while keeping incident irradiance 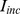 at 1000 µmol m^−2^ s^−1^. For the 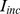 response curves, the photon flux densities were in a series: 0, 20, 50, 100, 150, 200, 500, 1000, 1500, 2000 µmol m^−2^ s^−1^, while keeping 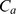 at 350 µmol mol^−1^ for measurements at 21% O_2_, and keeping 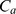 at 1000 µmol mol^−1^ for measurements at 2% O_2_ to ensure a non-photorespiration condition. The photosynthetic parameters of the FvCB model were estimated from these measurements [17] and are given in Table 4.

**Table 4:** Values (± standard error of estimate if applicable) of photosynthetic parameters estimated for flag leaves of wheat plants at flowering grown at low nitrogen (N) and high N levels at flowering stage. Estimates were made separately for photorespiratory (PR) and non-photorespiratory (NPR) conditions when necessary [17].

Parameters	High N	Low N
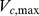 (µmolm^−2^ s^−1^)	65.8±0.8	58.5±0.8
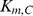 (μbar)	168±17	168±17
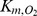 (mbar)	473	473
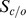 (mbar μbar^−1^)	3.13	3.13
*s*	0.380	0.403
Γ* (μbar)	34	34
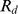 (µmol m^−2^ s^−1^) PR	1.317	0.939
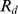 (µmol m^−2^ s^−1^) NPR	1.573	1.375
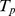 (µmol m^−2^ s^−1^)	12.9±0.13	11.1±0.19

### Definition of macroscale variables

The microscale model predicts local variables which may depend on the position inside the leaf, whereas the gas exchange and chlorophyll fluorescence experiments measure lumped, macroscale variables of the whole leaf. In order to compare both measurements and simulations, equivalent macroscale variables need to be calculated from the microscale simulation results. We will use the following convention for symbols: macroscopic variables which were estimated from gas exchange and chlorophyll fluorescence experiments are denoted by a ‘∧’ symbol. Volume averaged variables (area averaged variables in the 2-D model) calculated from the microscale model are overlined.

Chlorophyll fluorescence measurements can assess the photosystem II (PSII) electron transport efficiency as 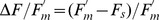 , where 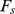 is the steady-state fluorescence, 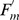 is the maximum fluorescence during a saturating light pulse [74]. Data for 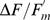 can be converted into the flux of potential electron transport (*J*) according to
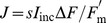
(12)where *s* is a calibration factor that can be estimated as the slope of the empirical linear relation between *A* and 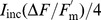 using data of non-photorespiratory measurements at 2% O_2_ combined with high CO_2_ levels (see Yin et al. [17], for more details). Using *J* estimated from the chlorophyll fluorescence measurements under photorespiration conditions, the mean mesophyll CO_2_ concentration 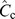 was estimated as [10]:
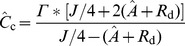
(13)where 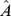 is the net CO_2_ assimilation rate based on the gas exchange measurements.

The volume averaged CO_2_ concentration of the mesophyll cell (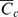) predicted by the microscale model was computed as
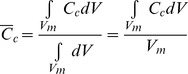
(14)The integration domain *V_m_* in Equation (14) is the volume (area in 2-D) of all mesophyll cells in the 2-D microstructural image of the leaf tissue.

On the basis of the assumption that *C_c_* can be reliably estimated by Equation (13) from combined gas exchange and chlorophyll fluorescence data, the mesophyll conductance 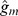 was calculated from [10]:
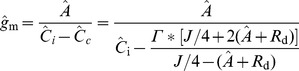
(15)where 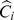 is the intercellular CO_2_ concentration from gas exchange measurements [7] and 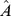 the measured photosynthesis rate. The equivalent whole-leaf 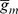 predicted by the microscale model is
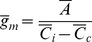
(16)where 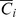 is the volume averaged intercellular CO_2_ concentration and computed from the microscale model according to a similar expression as in Equation (14). The whole leaf photosynthesis rate 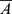 is calculated by integrating the CO_2_ flux from the epidermis to the ambient over the entire exchange surface.

### Model calibration and validation

The model equations were solved using the finite element environment Comsol Multiphysics vs. 3.5 (Comsol AB, Stockholm). The non-linear coupled model equations from (1) to (10) were discretized over the finite element mesh using the weak formulation [75]. The model equations were solved for steady-state conditions. Between the organelles, permeation through the membranes was taken into account. A direct solver was used for solving the resulting set of ordinary differential equations with relative tolerance less than 10^−6^.

Gas transport properties were obtained from the literature (Table 3). The photosynthetic parameters of the FvCB model for different N treatments and life stages were obtained from Yin et al.[17]. 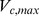 was estimated based on the chloroplastic CO_2_ concentration. The potential electron transport rate *J* was calculated from the chlorophyll fluorescence measurements (Equation 12). We assumed that all membranes had the same permeability (value indicated in Table 3), but because the chloroplast envelope is a double membrane we assigned half the permeability of the other (single) membranes to it.

For model calibration, data from experiments A1 and A2 of Table 5 were used. Using the photosynthesis response to ambient CO_2_ concentration (Yin et al. [17], the diffusivity values of the epidermis (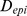) and of the cell wall (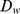) were estimated simultaneously by fitting the calculated CO_2_ concentration of the intercellular space and the mesophyll CO_2_ concentration determined from microscale model to the experimental data using a nonlinear least square estimation procedure in Matlab (The Mathworks, Inc., Natick, USA). The boundary condition used in the parameter estimation was 350 µmol mol^−1^ CO_2_ at 21% O_2_ while keeping 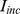 at 1000 µmol m^−2^ s^−1^ and 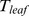 at 25°C. The resulting values were equal to 1.67×10^−7^ m^2^ s^−1^ and 3.437×10^−7^ m^2^ s^−1^ for 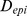 and 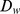, respectively (Table 3). Note that for reasons outlined before the stomata were not modelled explicitly but their conductance was implicitly included in 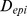. Irradiation affects stomatal aperture [34] and a significant effect on the measured stomatal conductance has been observed. Thus, for modelling of photosynthesis in response to irradiation, 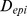 can be expected to vary with irradiance. For each measured light intensity, the corresponding 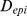 was therefore determined by fitting 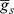 to 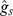 while keeping 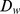 at the value determined previously.

**Table 5:** Description of data sets used in calibration and validation of model.

	Data set	Nitrogen (N) supply	Development stage	[CO_2_] (µmol mol^−1^)	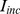 (µmol m^−2^ s^−1^)	O_2_ (%)	Experiments
Calibration	A1	High N	Flowering stage	350	1000	21	CO_2_ response curves
	A2	High N	Flowering stage	350	0, 20, 50, 100, 150, 200, 500, 1000, 1500, 2000	21	*I_inc_* response curves
Validation	A1	High N	Flowering stage	50, 100, 150, 200, 250, 500, 650, 1000, 1500	1000	2, 21	CO_2_ response curves
	B1	Low N	Flowering stage	50, 100, 150, 200, 250, 350, 500, 650, 1000, 1500	1000	2, 21	CO_2_ response curves
	C1	High N	2 weeks after flowering	50, 100, 150, 200, 250, 350, 500, 650, 1000, 1500	1000	2, 21	CO_2_ response curves
	D1	Low N	2 weeks after flowering	50, 100, 150, 200, 250, 350, 500, 650, 1000, 1500	1000	2, 21	CO_2_ response curves
	B2	Low N	Flowering stage	350	0, 20, 50, 100, 150, 200, 500, 1000, 1500, 2000	21	*I_inc_* response curves
	C2	High N	2 weeks after flowering	350	0, 20, 50, 100, 150, 200, 500, 1000, 1500, 2000	21	*I_inc_* response curves
	D2	Low N	2 weeks after flowering	350	0, 20, 50, 100, 150, 200, 500, 1000, 1500, 2000	21	*I_inc_* response curves

For validation, the model predictions were compared to experimental data that were not used for the parameter estimation, i.e. dataset B1, C1, D1, B2, C2 and D2 of Table 5. The same values of *D_epi_* and *D_w_* as in the calibration experiments were assumed.

## Supporting Information

Text S1 **Lumped microscale modeling.**
(DOC)

Figure S1
**Computed CO_2_ distribution in wheat leaf according to the model with and without chloroplasts.** The ambient conditions were 350 µmol mol^−1^ CO_2_, 21% O_2_, 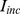 = 1000 µmol m^−2^ s^−1^ and 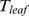 = 25°C. Concentrations are expressed in µmol m^−3^. (A) and (B) are simulation results with and without chloroplasts.(TIF)

Figure S2
**Simulations and measurements at different conditions of** 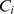 **at 21% O_2_,** 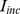 **= 1000 µmol m^−2^ s^−1^ and 25°C.** The left and right figures represent simulations at two weeks after flowering for high and low N supply flag leaves, respectively. Figures (A) and (B) show the net CO_2_ assimilation rate (*A*) as function of intercellular CO_2_ concentration 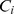. The symbols represent measurements (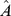 versus 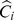) while the lines indicate model predictions (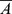 versus 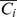). Figures (C) and (D) depict 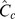 versus 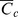. The diagonal lines indicate perfect correspondence. Figures (E) and (F) show 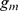 as function of 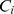. The solid (—) line represents 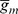 versus 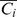. The symbols (o) represent the measured data (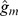 versus 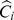). Data are from Yin et al. [17].(TIF)

Figure S3
**Model predictions (lines) versus measurements (symbols) of photosynthesis variables for 350 µmol mol^−1^ CO_2_, 21% O_2_,** 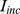 **from 0 to 2000 µmol m^−2^ s^−1^ and 25°C.** Left figures and right figures represent simulations for high N and low N supply flag leaves at two weeks after flowering. Figure (A) and (B) show 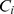 and 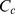 as function of 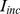; the solid lines (—) and dashed lines (- -) represent 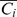 and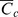, symbols (×) and (o) represent 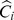 and 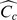, respectively. Figure (C) and (D) show *A* as function of 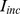, while figure (E) and (F) indicate the mesophyll conductance 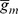 (—) or 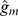 (o) as function of 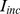. Data from Yin et al. [17].(TIF)

Figure S4
**Simulated net photosynthesis of wheat leaf as function of temperature.** (A) Temperature dependence of *V_c,max_* and *J_max_*. Values are normalized to 1 at 25°C. Arrhenius-like expressions for *V_c,max_* and *J_max_* as a function of temperature are described by [44] and [29], respectively. (B) Simulated net photosynthesis of wheat leaf as function of temperature. 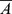, 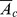 and 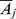 are the mean net photosynthesis rate, rubisco activity limited net photosynthesis rate and electron transport limited net photosynthesis rate computed from the microscale model. *V_c,max_* and *J_max_* as function of temperature are taken from [44] and [29], respectively while the temperature dependence of other FvCB parameters (*R_d_*, Γ*, 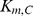, 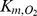) were was from [39] and [40]. Model predictions of photosynthesis were for high N wheat leaf at the flowering stage, 350 µmol mol^−1^ CO_2_, 21% O_2_, 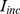 of 1000 µmol m^−2^ s^−1^.(TIF)
