# Supplementary material for: A Microscale Model for Combined CO2 Diffusion and Photosynthesis in Leaves
Source: PLoS One. 2012 Nov 7;7(11):e48376. doi: 10.1371/journal.pone.0048376 (PMC3492360; doi:10.1371/journal.pone.0048376)
Supplement: Text S1 — Lumped microscale modeling. (DOC) [file pone.0048376.s001.doc]

**Text S1. Lumped microscale modelling**

In the lumped microscale model, no distinction was made between mesophyll and vascular cells with respect to photosynthesis and gas exchange characteristics. The pH in the cell was assumed to be 7.8. Inside the mesophyll cells, the CO_2_ consumption by photosynthesis was modelled as a sink term including the net photosynthesis described by $-\left(A_{G}-R_{d}\right)/(d.f_{m})$ where *f_m_* is the volume fraction of the mesophyll cells to the leaf.

Simulation results showed that there was not much difference between the model with and without chloroplasts (Fig. S1) except of course inside the cells. The value of *g_m_* obtained with the model with vacuole and chloroplasts was 12.7% higher than that of the lumped model. Note that the lumped model did not contain chloroplasts and thus also no gas transport resistance due to the chloroplast envelope. Obviously, the intracellular CO_2_ gradients were very different while a similar gas concentration was observed in the pores for the lumped model and the model including chloroplasts.
