# Supplementary figures and images for: A Microscale Model for Combined CO2 Diffusion and Photosynthesis in Leaves
Source: PLoS One. 2012 Nov 7;7(11):e48376. doi: 10.1371/journal.pone.0048376 (PMC3492360; doi:10.1371/journal.pone.0048376)

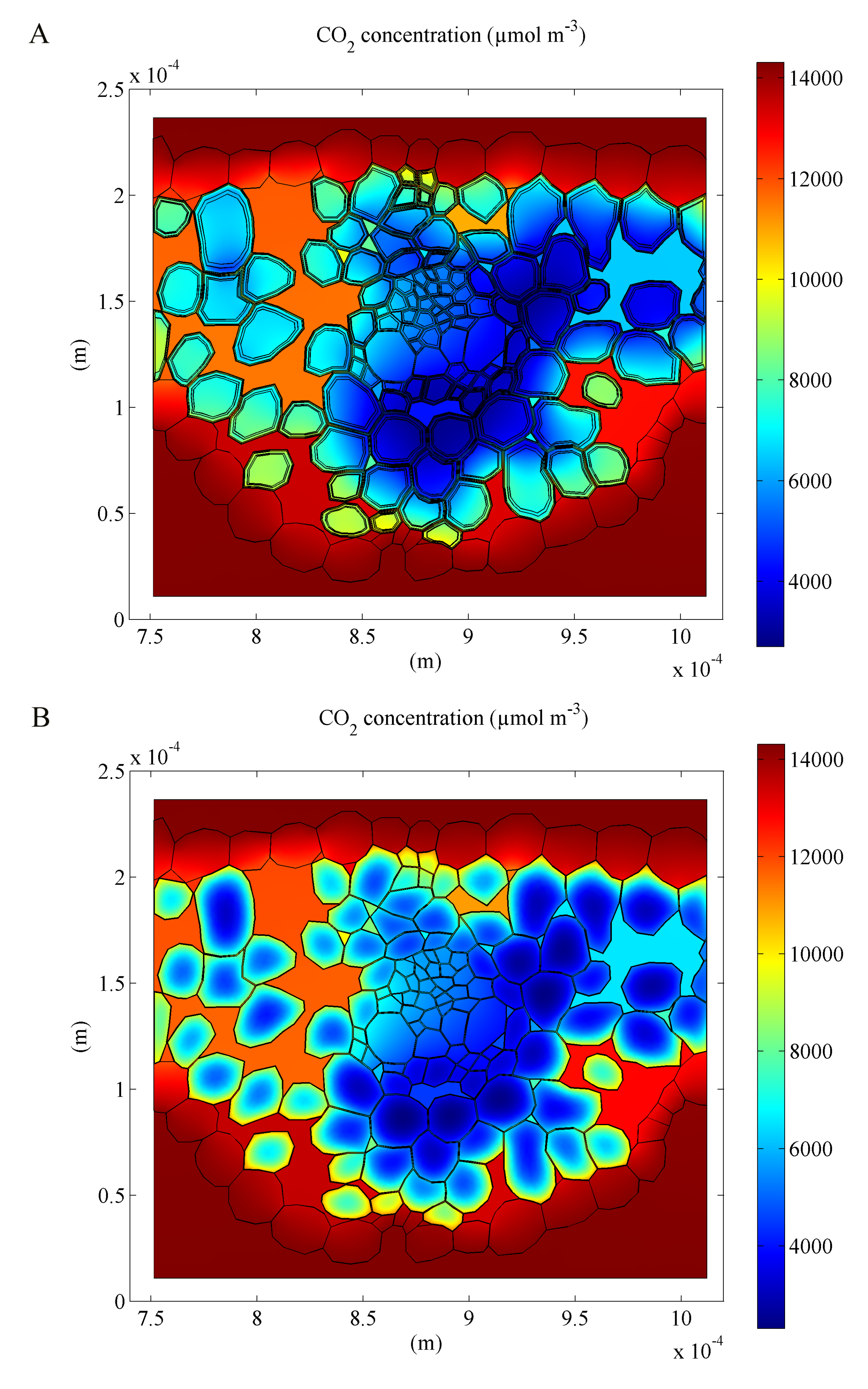

Supplement: Figure S1 — Computed CO2 distribution in wheat leaf according to the model with and without chloroplasts. The ambient conditions were 350 µmol mol−1 CO2, 21% O2, = 1000 µmol m−2 s−1 and = 25°C. Concentrations are expressed in µmol m−3. (A) and (B) are simulation results with and without chloroplasts. (TIF) [file pone.0048376.s002.tif]

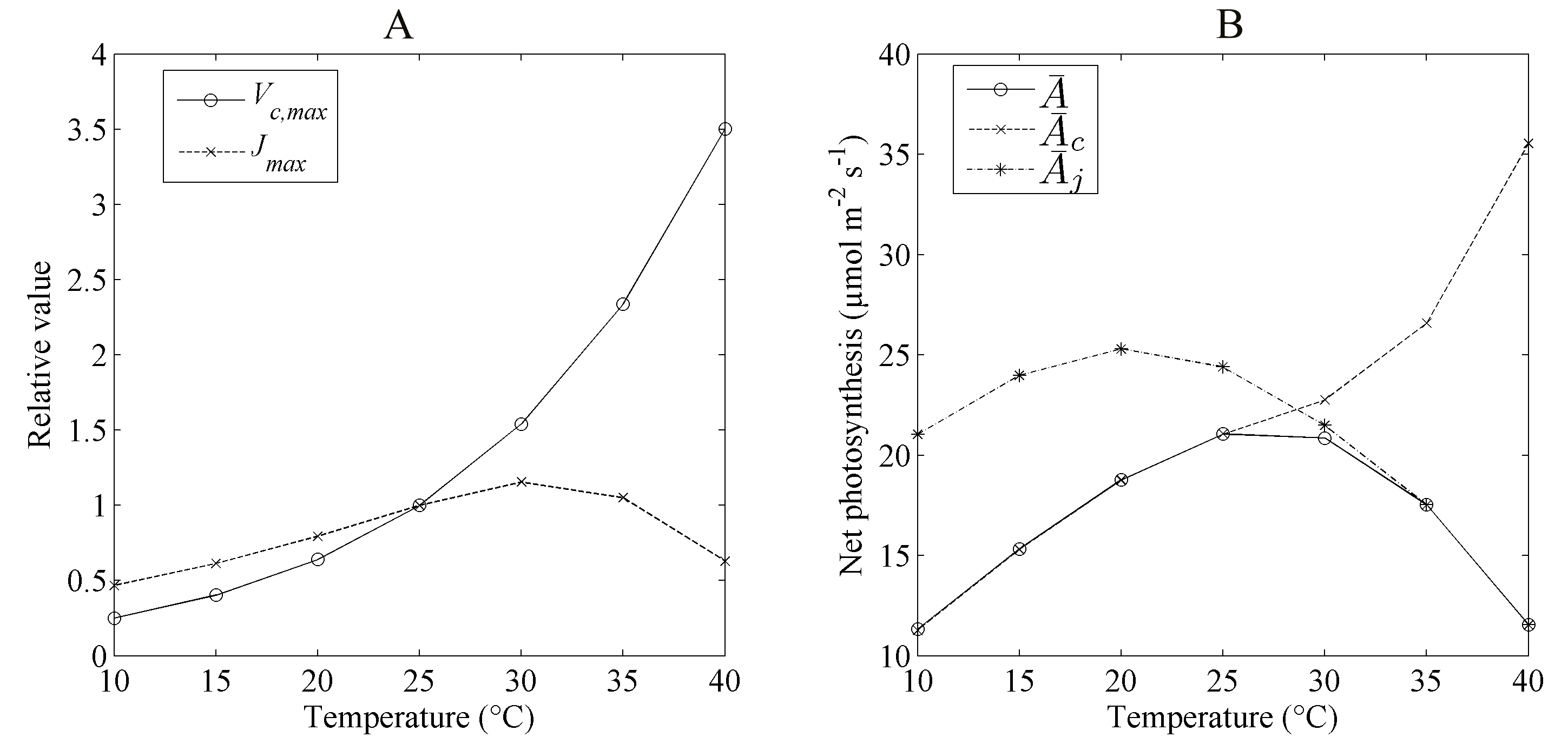

Supplement: Figure S4 — Simulated net photosynthesis of wheat leaf as function of temperature. (A) Temperature dependence of Vc,max and Jmax. Values are normalized to 1 at 25°C. Arrhenius-like expressions for Vc,max and Jmax as a function of temperature are described by [44] and [29], respectively. (B) Simulated net photosynthesis of wheat leaf as function of temperature. , and are the mean net photosynthesis rate, rubisco activity limited net photosynthesis rate and electron transport limited net photosynthesis rate computed from the microscale model. Vc,max and Jmax as function of temperature are taken from [44] and [29], respectively while the temperature dependence of other FvCB parameters (Rd, Γ*, , ) were was from [39] and [40]. Model predictions of photosynthesis were for high N wheat leaf at the flowering stage, 350 µmol mol−1 CO2, 21% O2, of 1000 µmol m−2 s−1. (TIF) [file pone.0048376.s005.tif]
